# Efficacy of tenofovir disoproxil fumarate at 240 weeks in patients with chronic hepatitis B with high baseline viral load

**DOI:** 10.1002/hep.26277

**Published:** 2013-05-03

**Authors:** Stuart C Gordon, Zahary Krastev, Andrzej Horban, Jörg Petersen, Jan Sperl, Phillip Dinh, Eduardo B Martins, Leland J Yee, John F Flaherty, Kathryn M Kitrinos, Vinod K Rustgi, Patrick Marcellin

**Affiliations:** 1 Henry Ford Health SystemDetroit, MI; 2 University HospitalSt. Ivan Rilsky, Sofia, Bulgaria; 3 Warsaw Medical UniversityWarsaw, Poland; 4 Liver Unit Asklepios Klinik St. GeorgHamburg, Germany; 5 Institute for Clinical and Experimental MedicinePrague, Czech Republic; 6 Gilead Sciences, Inc.Foster City, CA; 7 Metropolitan Liver DiseasesFairfax, VA; 8 Hôpital Beaujon, University of ParisClichy, France

## Abstract

We evaluated the antiviral response of patients with chronic hepatitis B (CHB) who had baseline high viral load (HVL), defined as having hepatitis B virus (HBV) DNA ≥9 log_10_ copies/mL, after 240 weeks of tenofovir disoproxil fumarate (TDF) treatment. A total of 641 hepatitis B e antigen (HBeAg)-negative and HBeAg-positive patients (129 with HVL) received 48 weeks of TDF 300 mg (HVL n = 82) or adefovir dipivoxil (ADV) 10 mg (HVL n = 47), followed by open-label TDF for an additional 192 weeks. Patients with confirmed HBV DNA ≥400 copies/mL on or after week 72 had the option of adding emtricitabine (FTC). By week 240, 98.3% of HVL and 99.2% of non-HVL patients on treatment achieved HBV DNA <400 copies/mL. Both groups had similar rates of histologic regression between baseline and week 240. Patients with HVL generally took longer to achieve HBV DNA <400 copies/mL than non-HVL patients, but by week 96, the percentages of patients with HBV DNA <400 copies/mL were similar in both groups. Among HVL patients, time to achieving HBV DNA <400 copies/mL was shorter among those initially receiving TDF, compared to ADV. No patient with baseline HVL had persistent viremia at week 240 or amino acid substitutions associated with TDF resistance. *Conclusion*: CHB patients with HVL can achieve HBV DNA negativity with long-term TDF treatment, although time to HBV DNA <400 copies/mL may be longer, relative to patients with non-HVL.

**C**hronic hepatitis B (CHB) patients with high pretreatment viral load (HVL) represent a clinical challenge. Higher levels of hepatitis B virus (HBV) DNA are associated with an increased risk for hepatocellular carcinoma (HCC) and cirrhosis.^1^ In addition, available evidence suggests that CHB patients with HVL are less likely to respond to interferon (IFN)-based regimens than those with lower viral load. In hepatitis B e antigen (HBeAg)-positive patients, having baseline HBV DNA <2 × 10^8^ IU/mL is a positive predictor of sustained response to pegylated interferon alpha (Peg-IFN-α).^4^ Furthermore, having HBV DNA levels ≤10^9^ copies/mL is predictive of HBeAg seroconversion with Peg-IFN-α2a treatment.^5^ Baseline HBV DNA level is also a predictor of response in HBeAg-negative patients treated with Peg-IFN-α2a with or without lamivudine.^6^

A similar trend with baseline HBV DNA level and virologic response (VR) has been observed with nucleos(t)ide analog treatment. Mean baseline HBV DNA has been indicated as a factor influencing likelihood of VR with entecavir therapy.^7^ In a study of treatment-naïve HBV patients who received entecavir for 3 years, 100% of patients with baseline HBV DNA <8 log_10_ copies/mL had undetectable HBV DNA (<12 IU/mL), whereas only 75% of patients with baseline HBV DNA ≥8 log_10_ copies/mL did.^8^ Of the patients who did not have HBV DNA undetectability, many had relatively low HBV DNA levels (≤3 log_10_ copies/mL), and it is not known whether these patients may have achieved further reductions in HBV DNA with longer treatment.

The nucleotide analog tenofovir disoproxil fumarate (TDF) was approved in 2008 for the treatment of CHB. In two phase III studies, which included a total of 641 patients, TDF suppressed viral replication in both HBeAg-positive and -negative patients.^9^ In these studies, 240 weeks of TDF-based therapy led to significant histologic regression, including reversal of cirrhosis in 74% of patients that had cirrhosis at baseline.^10^ Within this analysis, we examined whether HBeAg-positive and -negative patients with HVL, defined as pretreatment levels of HBV DNA ≥9 log_10_ copies/mL, responded as well to long-term TDF-based treatment as patients without pretreatment HVL (non-HVL).

## Patients and Methods

### Study Population

Patients included in the present analyses were participants in studies GS-US-174-0102 and GS-US-174-0103, which evaluated the use of TDF in CHB patients. The details of those studies have been described previously.^9^ Briefly, patients in both studies were randomly allocated (2:1) to receive TDF or adefovir dipivoxil (ADV) for 48 weeks. After completing 48 weeks for treatment, subjects were given the option of continuing with open-label TDF up to 384 weeks. On or after week 72, patients with confirmed HBV DNA ≥400 copies/mL were eligible to add emtricitabine (FTC) therapy at the investigator's discretion.

Patients were required to be hepatitis B surface antigen (HBsAg)^+^ at least 6 months before enrollment. Participants could not have a history of HCC or decompensation (ascites, jaundice, encephalopathy, or variceal hemorrhage) and needed to have a Knodell necroinflammatory score of ≥3 and fibrosis score <4 at the baseline liver biopsy, although a limited number of patients with cirrhosis were allowed in the study. Patients were required to have evidence of active HBV infection (i.e., detectable HBV DNA and elevated alanine aminotransferase [ALT]) and compensated liver disease.

Patients enrolled in Study 102 were either treatment naïve or experienced, were HBeAg negative and anti-HBe positive, and had HBV DNA >10^5^ copies/mL and ALT above the upper limit of normal (ULN), but ≤10 × ULN. Patients enrolled in Study 103 were HBeAg positive and treatment naïve (defined as <12 weeks nucleoside/nucleotide experience) and had HBV DNA >10^6^ copies/mL and ALT >2 × ULN, but ≤10 × ULN. All patients provided written consent, and the protocol was approved by the institutional review boards of the participating institutions.

Liver biopsies were performed at baseline, week 48, and week 240 and scored using the Knodell and Ishak scoring systems by a single independent pathologist blinded to patient outcomes. Cirrhosis was defined as Ishak scores of 5 or 6.

### Virologic Response (VR)

HVL was defined as having baseline HBV DNA ≥9 log_10_ copies/mL (8.24 log_10_ IU/mL). Non-HVL was defined as having baseline HBV DNA <9 log_10_ copies/mL. The primary endpoint was the proportion of HVL versus non-HVL patients having HBV DNA <400 copies/mL (69 IU/mL) at week 240 of treatment. Persistent viremia was defined as never having HBV DNA <400 copies/mL.

HBV DNA was measured using the Roche COBAS TaqMan (Roche Diagnotics, Branchburg, NJ) assay (lower limit of quantification [LLOQ]: 169 copies/mL or 29 IU/mL). HBsAg was quantified using the Abbott Architect Assay (Abbott Laboratories, Abbott Park, IL). Samples exceeding the upper limit of 250 IU/mL were diluted per the recommendations of the manufacturer (up to 1:999 dilution).

### Virologic Resistance

Virologic resistance was assessed annually by evaluating genotypic changes within the HBV polymerase/reverse transcriptase (pol/RT) in patients with HBV DNA ≥400 copies/mL who experienced virologic breakthrough or in the context of persistent viremia. Virologic breakthrough was defined as having either two consecutive HBV DNA values ≥400 copies/mL after having been <400 copies/mL or two consecutive HBV DNA values >1 log_10_ copies/mL from nadir. Conserved site changes or polymorphic site changes observed in >1 patient were confirmed by phenotyping, as previously described.^11^–^12^

### Statistical Analyses

For continuous variables, conventional descriptive statistics (n, mean, standard deviation, median, Q1, Q3, minimum, and maximum) were performed. Log_10_ transformation was used to transform certain continuous variables that were highly skewed (HBV DNA and quantitative HBsAg). A two-sided Wilcoxon rank-sum test was used to compare continuous variables between HVL and non-HVL patients. Categorical variables were summarized by number and percentage of patients that met the endpoint. A two-sided Mantel-Haenszel test was used to compare categorical variables between HVL and non-HVL patients. Analyses were performed with patients on treatment. Data for patients who added FTC were analyzed separately.

## Results

### Study Population

A total of 641 patients enrolled in GS-US-174-0102 (N = 375 patients) and GS-US-174-0103 (N = 266 patients) and received at least one dose of study drug. Of the 641 patients, 129 (20%) had HVL at baseline.

Median baseline HBV DNA levels were 9.52 log_10_copies/mL in the HVL group and 7.34 log_10_ in the non-HVL group (*P* < 0.001) (Table1). A greater proportion of patients with HVL were HBeAg^+^ at baseline (91.5% with HVL versus 28.9% with non-HVL; *P* < 0.001), and median age was lower in the HVL group. The proportion of patients with cirrhosis (Ishak 5/6) was comparable, with 18.6% in the HVL group and 25.2% in the non-HVL group (*P* = 0.148). Distribution of viral genotypes was not significantly different between the HVL and non-HVL groups (*P* = 0.292).

**Table:** Baseline Clinical and Demographic Characteristics

	HVL* (n = 129)	Non-HVL* (n = 512)	*P* Value†
Median (IQR) age, years	31 (23, 39)	43 (33, 50)	<0.001
Sex, n (%)			
Male	96 (74.4)	377 (73.6)	0.856
Female	33 (25.6)	135 (26.4)	
Median (IQR) HBV DNA,	9.52 (9.25, 9.73)	7.34 (6.23, 8.29)	<0.001
log_10_ copies/mL			
HBeAg positive at baseline, n (%)	118 (91.5)	148 (28.9)	<0.001
Anti-HBeAg positive at baseline, n (%)	13 (10.2)	375 (73.2)	<0.001
Previous LAM/FTC experience	6 (4.7)	69 (13.5)	0.005
> 12 weeks, n (%)			
No. (%) with cirrhosis (Ishak 5/6)	24 (18.6)	128 (25.2)	0.148
HBV genotype (%)			
A	28 (22.0)	75 (15.0)	0.292
B	12 (9.4)	62 (12.4)	
C	19 (15.0)	93 (18.6)	
D	62 (48.8)	253 (50.5)	
Other‡	6 (4.7)	18 (3.6)	

Abbreviation: IQR, interquartile range.

* HVL refers to HBV DNA ≥9 log_10_ copies/mL. Non-HVL refers to HBV DNA <9 log_10_ copies/mL.

† For categorical data, two-sided Cochran-Mantel-Haenszel tests were used. For continuous data, two-sided Wilcoxon rank-sum tests were used.

‡ Other includes genotypes E-H. Missing and samples that could not be evaluated were excluded.

### VR

**Figure 1:**
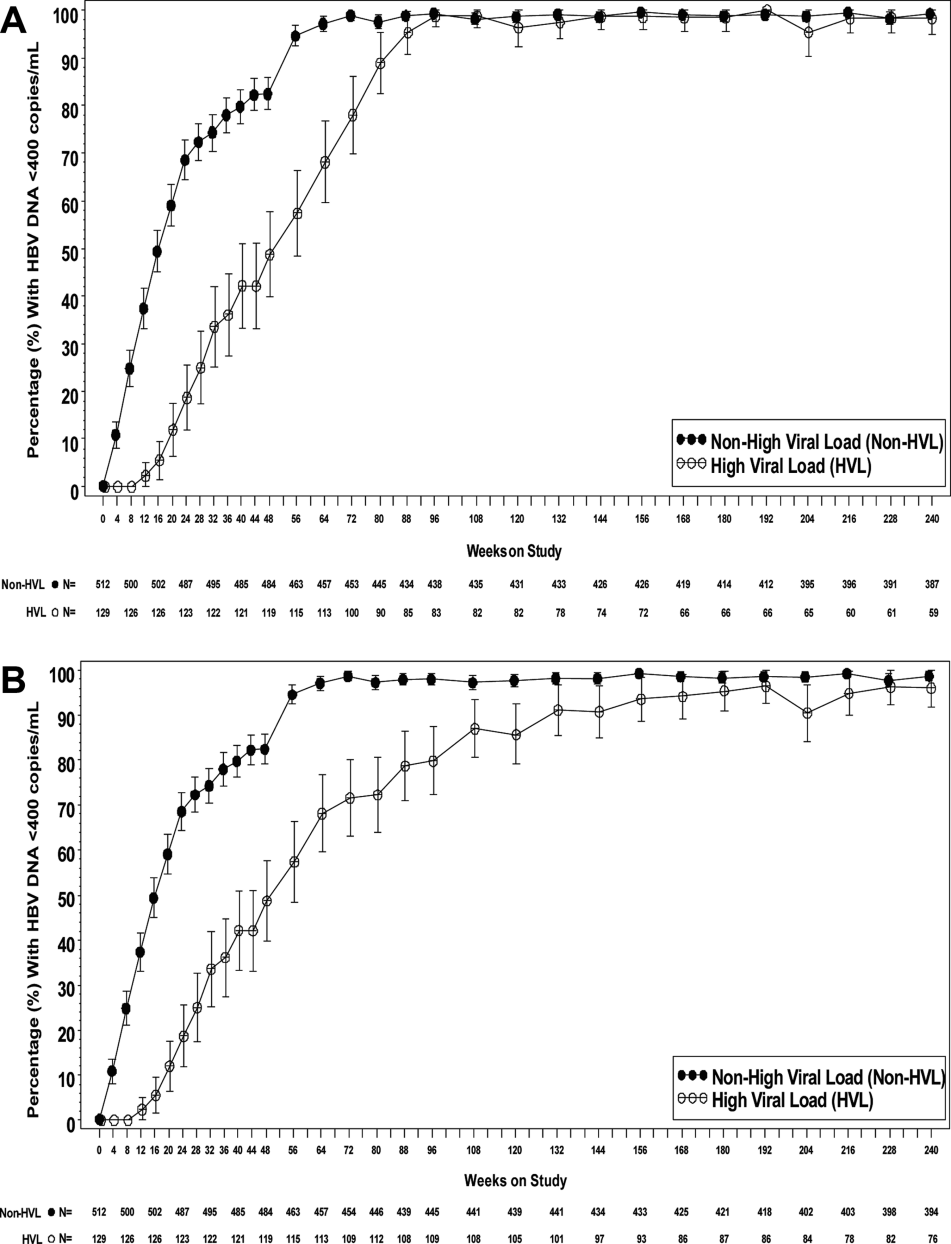
Time to viral negativity on the basis of baseline viral load. The proportion of patients with HBV high baseline viral load (≥9 log_10_ copies/mL) and non-high baseline viral load (<9 log_10_ copies/mL) who achieved HBV DNA <400 copies/mL during TDF long-term treatment. (A) Excludes patients who received FTC. (B) Includes patients who received FTC. Vertical bars represent 95% confidence intervals.

**Figure 2:**
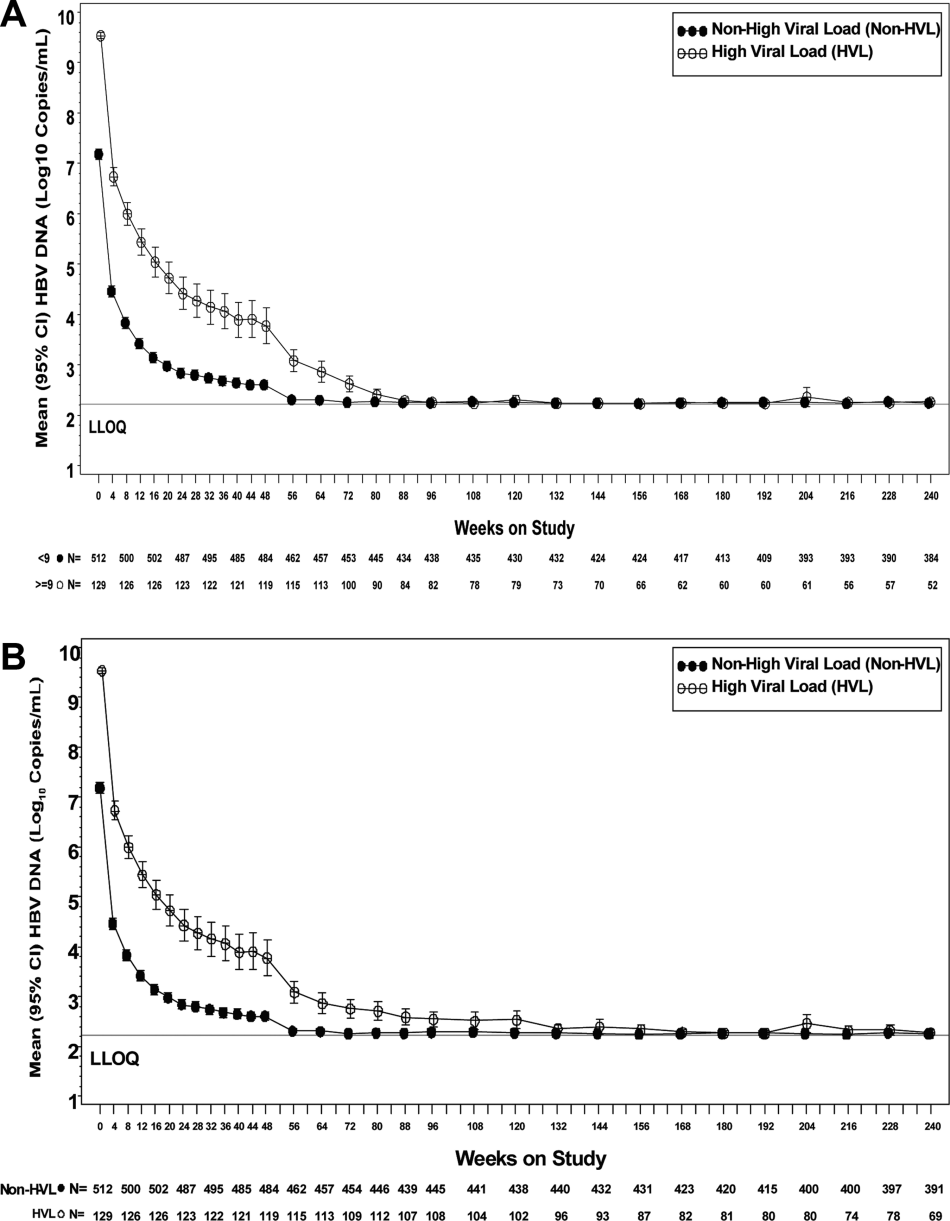
Viral load over time. Mean HBV DNA for patients with high baseline viral load (≥9 log_10_ copies/mL) and non-high baseline viral load (<9 log_10_ copies/mL) during TDF long-term treatment. (A) Excludes patients who received FTC. (B) Includes patients who received FTC. Vertical bars represent 95% confidence intervals.

**Figure 3:**
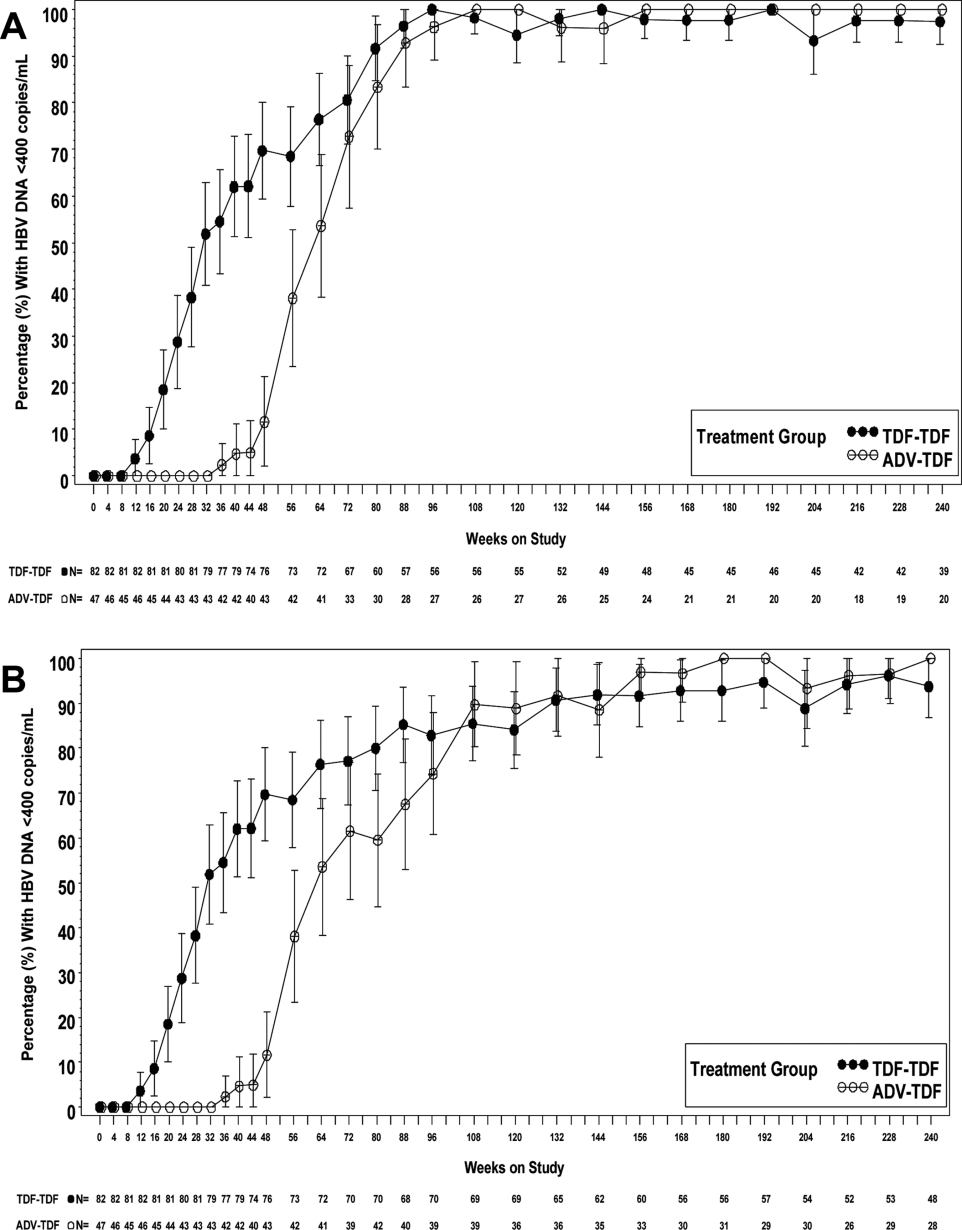
Time to viral negativity on the basis of initial therapy. The proportion of patients with HBV high baseline viral load who reached HBV DNA <400 copies/mL after initial treatment with either TDF or ADV. (A) Excludes patients who received FTC. (B) Includes patients who received FTC. Vertical bars represent 95% confidence intervals.

A total of 489 patients completed 240 weeks of therapy (304 from GS-US-174-0102 and 185 from GS-US-174-0103) (Table2 and Supporting Table1). On-treatment VR was high for both HVL and non-HVL patients, with 98.3% of HVL and 99.2% of non-HVL patients achieving HBV DNA <400 copies/mL by week 240 (Fig. 1). Also, by week 240, HBV DNA levels were <169 copies/mL for 96.6% of HVL patients and for 99.0% of non-HVL patients on treatment. The time course for achieving HBV DNA <400 copies/mL was longer among HVL patients versus non-HVL patients (Fig. 2), but by week 96, the percentage of patients with HBV DNA <400 copies/mL was similar between the two groups. At week 48, the percentage of HVL patients achieving HBV DNA <400 copies/mL was higher among patients initially randomized to TDF, compared to ADV (Fig. 3); however, after the switch to TDF, the rate of virologic suppression rapidly increased, and by week 96, the proportion of patients with HBV DNA <400 copies/mL was similar in both groups.

**Tabel 2:** Disposition of Patients at Week 240

	HVL* (n = 129)	Non-HVL† (n = 512)	Total (n = 641)
Completed 240 weeks, n (%)	83 (64.3)	406 (79.3)	489 (76.3)
Discontinued before week 240, n (%)	46 (35.7)	106 (20.7)	152 (23.7)
Declined extension	0	4 (0.8)	4 (0.6)
Investigator discretion	3 (2.3)	7 (1.4)	10 (1.6)
Lost to follow-up	13 (10.1)	24 (4.7)	37 (5.8)
Protocol violation	2 (1.6)	5 (1.0)	7 (1.1)
Safety, tolerability, or efficacy	5 (3.9)	17 (3.3)	22 (3.4)
Seroconversion	6 (4.7)	4 (0.8)	10 (1.6)
Study discontinued by sponsor	1 (0.8)	0	1 (0.2)
Withdrew consent	16 (12.4)	45 (8.8)	61 (9.5)
HBV DNA <400 copies/mL at the last visit before discontinuation, n (%)	28/46 (60.9)	77/106 (72.6)	105/152 (69.1)

* HVL, pretreatment HBV DNA ≥9 log_10_ copies/mL.

‡ Non-HVL, pretreatment HBV DNA <9 log_10_ copies/mL.

Among patients HBeAg^+^ at baseline, HBsAg loss was higher among those with HVL, compared with non-HVL patients (Table3), with 19.3% among those with HVL and 4.3% in the non-HVL group (*P* < 0.001). A similar pattern was observed for anti-HBsAg seroconversion, with 13.6% among HVL and 4.3% among non-HVL patients (*P* = 0.011).

**Table:** Week 240 On-Treatment Clinical Characteristics*

Characteristic	HVL† (%)	Non-HVL† (%)	*P* Value‡
ALT normalization	41/59 (69.5)	303/363 (83.5)	0.010
HBsAg loss§	17/88 (19.3)	6/141 (4.3)	<0.001
HBsAg seroconversion§>	12/88 (13.6)	6/141 (4.3)	0.011
HBeAg loss§	23/47 (48.9)	56/97 (57.7)	0.322
HBeAg seroconversion§	18/47 (38.3)	46/96 (47.9)	0.279
Cirrhosis (Ishak 5/6)	1/47 (2.1)	27/285 (9.5)	0.093
Persistent viremia∥	0	0	—

* Excludes patients who added emtricitabine.

† HVL, pretreatment HBV DNA ≥9 log_10_ copies/mL. Non-HVL, pretreatment HBV DNA <9 log_10_ copies/mL.

‡ For categorical data, two-sided Cochran-Mantel-Haenszel tests were used. For continuous data, two-sided Wilcoxon rank-sum tests were used.

§ For patients HBeAg positive at baseline.

∥ Persistent viremia defined as never having HBV DNA <400 copies/mL.

Thirty-five HVL patients were eligible to add FTC between weeks 72 and 240. One patient, who was not eligible to add FTC per study criteria, added FTC at the discretion of the investigator. Of the 35 eligible patients, 28 switched to FTC/TDF and 7 remained on TDF monotherapy. Adding FTC did not appear to affect HBV DNA outcomes in HVL patients, because 66% (19 of 29) on FTC/TDF and 86% (6 of 7) on TDF had HBV DNA <400 copies/mL at week 240 or last visit.

### Virologic Breakthrough

No patient remaining in the study at week 240 had persistent viremia. Among HVL patients, 12 experienced a total of 13 episodes of viral breakthrough (patient 9 had two independent episodes); all patients with viral breakthrough were HBeAg^+^ at baseline. Eight episodes of breakthrough occurred during TDF monotherapy and five occurred during FTC/TDF combination therapy. Genotypic analyses of the HBV pol/RT revealed that 7 patients had no change from baseline (observed for both episodes of virologic breakthrough in patient 9), 3 had unique polymorphic changes, and 2 had conserved site changes (Table4). Analysis of TDF plasma levels and pill counts suggested that 8 of 13 (62%) episodes of virologic breakthrough occurred when patients were nonadherent to study medication. *In vitro* phenotypic analyses of the 2 patients with conserved site changes (1 and 11; rtG152E and rtK168N, respectively) revealed no evidence of reduced susceptibility to TDF.

**Table:** Summary of Genotypic and Phenotypic Results for HVL Patients Who Experienced Virologic Breakthrough

Patient	Treatment	Breakthrough Week	HBV pol/RT Sequence Changes*	Phenotyping Result†	Treatment Adherent?‡
1	ADV-TDF	132	**rtG152E**	1.8	Yes
2	ADV-TDF	64	None	Not tested	No
3	ADV-TDF	144	rtT213S/T	Not tested	No
4	ADV-TDF-FTC/TDF	120	None	1	Yes
5	TDF	36	rtV142E/V	ND	Yes
6	TDF	44	None	ND	Yes
7	TDF	80	None	1	No
8	TDF	72	None	1	No
9a	TDF	32	None	0.9	No
9b	TDF-FTC/TDF	108	None	Not tested	No
10	TDF-FTC/TDF	228	None	1.1	Yes
11	TDF-FTC/TDF	204	rtR138K, **rtK168N**, rtT212K	0.9	No
12	TDF-FTC/TDF	132	rtN139K/N	ND	No

Patient 9 had two independent episodes of virologic breakthrough.

Abbreviation: ND, not able to be determined.

* Conserved site changes are noted in bold.

† Phenotyping results are the 50% effective concentration (EC_50_) fold change value, which is the EC_50_ value of the on-treatment sample, compared to the EC_50_ value of the baseline sample. The interassay variability for TDF susceptibility is <2 fold of the mean EC_50_ values. Three patients (5, 6, and 12) were not tested because polymerase chain reaction amplification with phenotyping primers failed.

‡ Treatment adherence was determined by measuring TDF levels in plasma.

## Discussion

Data from this 240-week study of the nucleotide analog, TDF, suggest that CHB patients with HVL can achieve VR at similar rates as patients with lower viral loads, but VR tends to take longer in HVL patients. Previous studies of the nucleoside analog, entecavir, suggested that HVL was a negative predictor of response, defined either as HBV DNA <400 copies/mL during on-treatment follow-up or HBV DNA being undetectable at year 3 of treatment^7^–^8^; however, the analyses were conducted in smaller patient populations treated for shorter time courses. In this study, by the end of 240 weeks, neither persistent viremia nor TDF resistance were observed in any patient, regardless of baseline viral level.

During the first 48 weeks of therapy, HBV DNA declines in a biphasic pattern (Fig. 2) similar to what has been reported on previously with ADV therapy.^13^ In the HVL group, HBV DNA continued to decline from week 48 through 88, whereas, in the non-HVL group, many patients achieved HBV DNA <400 copies/mL by week 56. By week 96, the proportions of patients who had HBV DNA <400 copies/mL was similar between the groups.

Our results are in contrast with perceptions in the clinical setting that HVL patients are challenging to treat. These perceptions have been, at least in part, caused by experience with earlier antivirals, such as lamivudine and telbivudine, to which some patients had minimal or no VR and others experienced breakthrough. With lamivudine monotherapy, 76% of patients develop resistance after 5 years, and with telbivudine, approximately 25% of HBeAg^+^ and 11% of HBeAg^−^ patients had resistance after 2 years.^14^ ADV, though effective against lamivudine- and telbivudine-resistant mutants, has limited potency and is generally used as add-on therapy.^14^

Given the concern for development of resistance with earlier antivirals, this study of TDF monotherapy was designed such that FTC could be added to patients with HBV DNA ≥400 copies/mL at 72 weeks. However, add-on therapy with FTC appeared to be unnecessary, even in patients with HVL, because it did not provide further benefit over TDF alone regarding VR or suppression of resistance. In a recent study, combination entecavir and TDF therapy conferred an incremental benefit over entecavir monotherapy for reaching HBV DNA <50 IU/mL in HBeAg-positive patients with high baseline viral loads after 96 weeks of treatment.^15^ Our results suggest that TDF monotherapy is effective in patients with high baseline viral levels, with 98.3% of HVL and 99.2% of non-HVL patients achieving HBV DNA <400 copies/mL and no persistent viremia detected by week 240.

Rate of ALT normalization was high in both HVL and non-HVL groups, although the rate was higher in the non-HVL group. The majority of patients in both HVL and non-HVL groups had regression of histological cirrhosis. HBsAg loss and HBsAg seroconversion were higher among patients with HVL versus non-HVL. No obvious differences in patient or disease characteristics accounted for the differential rates of HBsAg loss and seroconversion. More research is needed to determine whether and how viral load might affect HBsAg loss and seroconversion in patient populations outside of this study.

From the outset of HBV antiviral therapy, it has been conventionally accepted that patients with lower baseline viral levels and higher baseline serum ALT values are more responsive than patients with higher viral levels and/or lower ALT values. In 2010, Wu et al.^16^ found that among HBeAg-negative patients, those with minimally raised serum ALT values had similar antiviral responsiveness as patients with higher values. In the present analysis, we have explored the prognostic significance of baseline serum HBV DNA levels; our finding of equal virologic responsiveness, regardless of baseline viral load, now confirms that these earlier perceptions are no longer applicable in the current antiviral arena.

This study only included patients in the immune clearance phase of HBV infection, and therefore the results cannot be extrapolated to immunotolerant patients, who likewise generally have HVL, but who do not have elevated ALT, and therefore may have little evidence of histologic inflammation. The effectiveness of TDF and TDF plus FTC is currently being evaluated in CHB patients with normal ALT levels. Additionally, the normal ALT levels associated with the immunotolerant population make direct analyses of ALT and comparisons between the two groups difficult.

In conclusion, our results suggest that long-term TDF monotherapy can lead to virologic suppression in the vast majority of CHB patients with HVL, regardless of HBeAg status. The extent of virologic suppression and absence of resistance with TDF in this study is indicative of a shifting HBV treatment landscape in which risk of nonresponse or development of resistance is declining with the adoption of newer, more-potent antivirals. Accordingly, HVL should not be a deterrent for treatment. For patients with CHB with HVL treated with TDF, our findings offer the reassurance that the long-term VR should prove excellent.

## Abbreviations

ADV: adefovir dipivoxil
ALT: alanine aminotransferase
CHB: chronic hepatitis B
FTC: emtricitabine
HBeAg: hepatitis B e antigen
HBV: hepatitis B virus
HBsAg: hepatitis B surface antigen
HCC: hepatocellular carcinoma
HVL: high viral load
IFN: interferon
LLOQ: lower limit of quatification
Peg-IFN-α: pegylated interferon alpha
pol/RT: HBV polymerase/reverse transcriptase
TDF: tenofovir disoproxil fumarate
ULN: upper limit of normal
VR: virologic response
