# Supplementary figures and images for: Efficacy of tenofovir disoproxil fumarate at 240 weeks in patients with chronic hepatitis B with high baseline viral load
Source: Hepatology. 2013 May 3;58(2):505–13. doi: 10.1002/hep.26277 (PMC3842114; doi:10.1002/hep.26277)

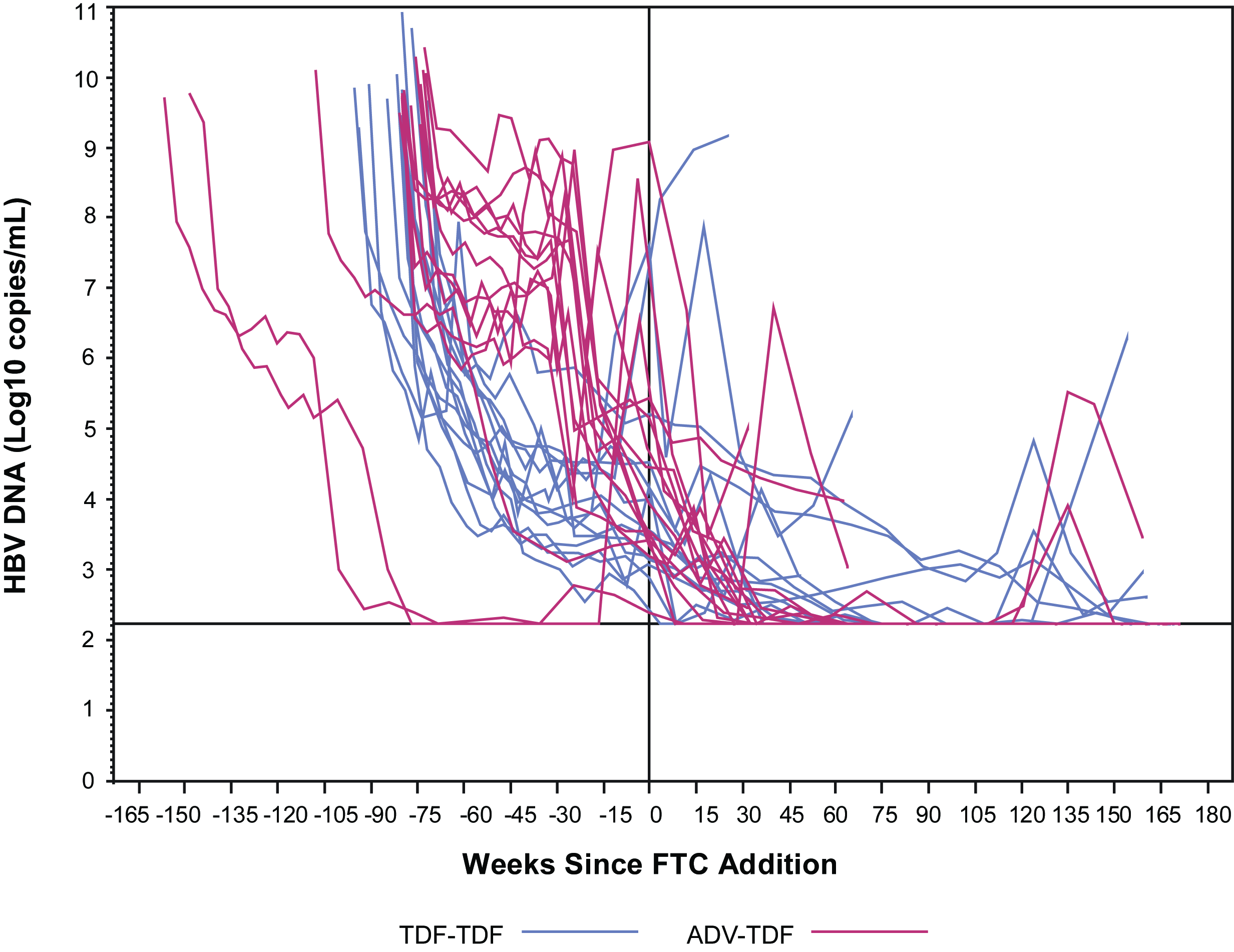

Supplement: Supplementary file 1 [file hep0058-0505-sd1.tif]
