# Supplementary material for: Efficacy of tenofovir disoproxil fumarate at 240 weeks in patients with chronic hepatitis B with high baseline viral load
Source: Hepatology. 2013 May 3;58(2):505–13. doi: 10.1002/hep.26277 (PMC3842114; doi:10.1002/hep.26277)
Supplement: Supplementary file 2 [file hep0058-0505-sd2.doc]

**Supplementary Table 1. Baseline Clinical and Demographic Characteristics Among 489 Subjects Who Completed 240 Weeks of Therapy.**

|  | **High Viral Load^a^**  **(n=83)** | **Non-High Viral Load^a^ (n=406)** | **p-value^b^** |
| --- | --- | --- | --- |
|  |  |  |  |
| **Median (IQR) age, years** | 33 (23, 43) | 43 (36, 51) | <0.001 |
|  |  |  |  |
| **Sex, n (%)** |  |  |  |
| **Female** | 15 (18.1) | 93 (22.9) | 0.334 |
| **Male** | 68 (81.9) | 313 (77.1) |  |
|  |  |  |  |
| **Median (IQR) HBV DNA,** | 9.5 (9.3, 9.8) | 7.3 (6.2, 8.3) | <0.001 |
| **log_10_ copies/mL** |  |  |  |
|  |  |  |  |
| **HBeAg-positive at baseline, n (%)** | 74 (89.2) | 111 (27.3) | <0.001 |
|  |  |  |  |
| **Anti-HBeAg-positive at baseline, n (%)** | 11 (13.3) | 303 (74.6) | <0.001 |
|  |  |  |  |
| **Previous LAM/FTC experience** | 5 (6.0) | 58 (14.3) | 0.041 |
| **> 12 weeks, n (%)** |  |  |  |
|  |  |  |  |
| **No. (%) with cirrhosis (Ishak 5/6)** | 16 (19.8) | 107 (26.5) | 0.204 |
|  |  |  |  |
| **HBV genotype n (%)** |  |  |  |
| **A** | 19 (23.5) | 61 (15.4) | 0.067 |
| **B** | 3 (3.7) | 51 (12.9) |  |
| **C** | 15 (18.5) | 66 (16.7) |  |
| **D** | 40 (49.4) | 206 (52.2) |  |
| **Other^c^** | 4 (4.9) | 11 (2.8) |  |

^a^ High viral load (HVL) refers to hepatitis B virus HBV DNA ≥ 9 log_10_ copies/mL. Non high-viral-load (non-HVL) refers to HBV DNA < 9 log_10_ copies/mL.

^b^For categorical data, two-sided Cochran-Mantel-Haenszel tests were used. For continuous data, two‑sided Wilcoxon Rank Sum tests were used.

^c^Other includes genotypes E-H. Missing and samples that could not be evaluated were excluded.

**Supplementary Table 2. Clinical Characteristics at Week 240^a^**

|  | **High Viral Load^b^** | **Non-High Viral Load^b^** | **p-value^c^** |
| --- | --- | --- | --- |
| HBV DNA < 400 copies/mL | 73/76 (96.1) | 389/394 (98.7) | 0.099 |
| ALT normalization | 54/77 (70.1) | 303/369 (82.9) | 0.010 |
| HBeAg loss^d^ | 24/64 (37.5) | 57/101 (56.4) | 0.018 |
| HBeAg seroconversion^d^ | 19/64 (29.7) | 47/100 (47.0) | 0.028 |
| HBsAg loss^d^ | 17/117 (14.5) | 6/146 (4.1) | 0.003 |
| HBsAg seroconversion^d^ | 12/117 (10.3) | 6/146 (4.1) | 0.050 |
| Cirrhosis (Ishak 5/6) | 1/59 (1.7) | 27/291 (9.3) | 0.051 |
| Persistent viremia^e^ | 0 | 0 | --- |

^a^ Includes patients who added emtricitabine.

^b^ High viral load, pretreatment hepatitis B virus (HBV) DNA ≥ 9 log_10_ copies/mL. Non high-viral load, pretreatment HBV DNA < 9 log_10_ copies/mL.

^c^ For categorical data, two-sided Cochran-Mantel-Haenszel tests were used. For continuous data, two-sided Wilcoxon Rank Sum tests were used.

^d^ For patients HBeAg-positive at baseline.

^e^ Persistent viremia defined as never having HBV DNA < 400 copies/mL.
